# ICP4-induced miR-101 attenuates HSV-1 replication

**DOI:** 10.1038/srep23205

**Published:** 2016-03-17

**Authors:** Xiangling Wang, Caifeng Diao, Xi Yang, Zhen Yang, Min Liu, Xin Li, Hua Tang

**Affiliations:** 1 Tianjin Life Science Research Center and Department of Pathogen Biology, School of Basic Medical Sciences, Tianjin Medical University, 22 Qi-Xiang-Tai Road, Tianjin 300070, China

## Abstract

Hepes simplex Virus type 1 (HSV-1) is an enveloped DNA virus that can cause lytic and latent infection. miRNAs post-transcriptionally regulate gene expression, and our previous work has indicated that HSV-1 infection induces miR-101 expression in HeLa cells. The present study demonstrates that HSV-1-induced miR-101 is mainly derived from its precursor hsa-mir-101-2, and the HSV-1 immediate early gene ICP4 (infected-cell polypeptide 4) directly binds to the hsa-mir-101-2 promoter to activate its expression. RNA-binding protein G-rich sequence factor 1 (GRSF1) was identified as a new target of miR-101; GRSF1 binds to HSV-1 p40 mRNA and enhances its expression, facilitating viral proliferation. Together, ICP4 induces miR-101 expression, which downregulates GRSF1 expression and attenuates the replication of HSV-1. This allows host cells to maintain a permissive environment for viral replication by preventing lytic cell death. These findings indicate that HSV-1 early gene expression modulates host miRNAs to regulate molecular defense mechanisms. This study provides novel insight into host-virus interactions in HSV-1 infection and may contribute to the development of antiviral therapeutics.

Herpes simplex virus type 1 (HSV-1) is a linear double-stranded DNA virus that, as a major human pathogen, mainly infects epithelial and neuronal cells, causing a variety of potentially fatal diseases^1^. HSV-1 has two distinct infection phases: productive (“lytic”) infection and latent infection. During the lytic process, HSV-1 expresses approximately 80 proteins, which are transcribed under a strictly regulated cascade of three gene types: immediate early genes (IE), early genes (E) and late genes (L)^2^. Infected-cell polypeptide 4 (ICP4) is one of the major regulatory factor that is required to efficiently activate the transcription of early and late viral genes during HSV infection^3^,^4^,^5^. ICP4 is a large, complex molecule that exists in cells as a 350 kDa dimer^6^. Its hydrodynamic properties indicate that it is very elongated in shape^7^,^8^, the ability may provide ICP4 the potential function as a transactivator over long distances^9^. For instance, ICP4 binds to the proximal human vascular endothelial growth factor (VEGF)-A promoter and sufficient to promote VEGF-A transcription; this process requires a tract of GC-rich sequences in the VEGF-A promoter, which is similar to the promoters for the HSV-1 E genes that are normally transactivated by ICP4. Therefore, ICP4 can activate both the VEGF-A promoter and HSV-1 E gene expression^10^. During latent infection, HSV-1 can establish permissive environment in host cells for HSV-1 replication to promote persistent infection in certain time by viral mutation and viral tropism and even by regulating antiviral factors of host cells.

Viruses have evolved mechanisms to regulate and escape host antiviral activity^11^, and complexly regulated by a variety of host factors, including retinoic acid inducible gene-1 (RIG-1), interferon (IFN), cyclooxygenase II (COX2), RNA-binding protein G-rich sequence factor 1 (GRSF1), and interferon stimulated genes (ISGs)^12^,^13^,^14^,^15^. GRSF1 belongs to a family of RNA-binding proteins called the heterogeneous nuclear ribonucleoprotein F/H protein family (hnRNP F/H)^16^, which includes hnRNP F, hnRNP H, hnRNP H3, hnRNP H2 and GRSF1^17^. HnRNP F/H proteins have been shown to specifically interact with guanine-rich (G-rich) stretches of RNA via quasi-RNA recognition domains (qRRMs)^18^. The cis-acting RNA element for GRSF1 consists of a G-rich stretch of RNA. GRSF1 has been implicated in influenza infection, embryonic brain development and the regulation of apoptosis^14^.

miRNAs are endogenous ~23 nt RNAs that bind to 3′UTRs of target mRNAs to regulate their expression^19^, and involved in various biological processes including virus-host interaction^20^,^21^,^22^. Viral infection generally results in dramatic changes in cellular mRNA expression including the pattern of cellular miRNA expression^23^, which represents a disastrous event in the life of host cells. For instance, the liver-specific cellular miR-122 is essential for hepatitis C virus (HCV) replication by interacting with 5′UTRs in the HCV genome^24^. miR-199a-3p and miR-210 bind to the HBsAg coding region and the pre-S1 region of hepatitis B virus (HBV) transcripts to suppress HBV proliferation^25^. Except for sequence-specific binding manner miRNAs also can modulate the host transcriptome indirectly to generate permissive environment for virus replication^26^. As reported, miR-132 induced by HSV-1 and human cytomegalovirus (HCMV) infection and negatively regulates the expression of interferon-stimulated genes to enhance viral replication^27^. However, little is known about the mechanism of virus-modulated host miRNA expression. And how these miRNAs affect the process of viral infection largely remains unclear. Our previous study indicated that miR-101 is highly induced in the early stages of HSV-1 infection, and miR-101 is involved in HSV-1 replication^22^; however, the mechanism of miR-101 upregulation and its suppression of HSV-1 replication remain unknown.

The miR-101 transcript includes two precursors: hsa-miR-101-1 and hsa-mir-101-2 which located on chromosome 1 and chromosome 9 respectively. Here, we demonstrate that HSV-1 mainly induced the expression of hsa-mir-101-2 but not hsa-mir-101-1. Chromatin immunoprecipitation (ChIP) and electrophoretic mobility shift assays (EMSA) revealed that HSV-1 ICP4 induced hsa-mir-101-2 expression by directly binding to its promoter region. Furthermore, GRSF1 which is a novel target of miR-101 was found to promote HSV-1 replication by binding to HSV-1 mRNA to facilitate its translation. Thus, HSV-1-induced miR-101 decreases GRSF1 expression and represses HSV-1 replication, which may be required to establish a suitable environment for HSV-1 replication by suppressing host cells death. These findings provide novel insights into the mechanisms underlying the interaction between viruses and host cells.

## Results

### miR-101 is induced by HSV-1 infection

**Figure 1:**
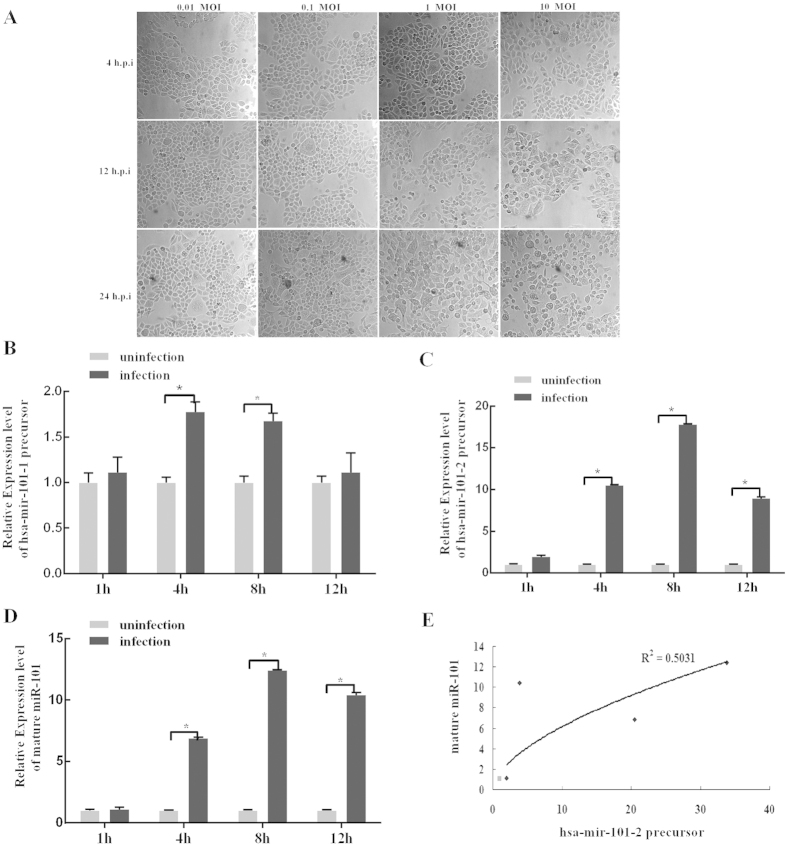
The expression of miR-101 is induced by HSV-1 infection. (**A**) The difference cytopathic effect (CPE) of HeLa cells infected with HSV-1 at different virus titer and time. HeLa cells were infected with HSV-1 at a MOI of 0.1, 1 or 10, and CPE was monitored at the indicated time points. Magnification, 200×. (**B–D**) HeLa cells were infected by HSV-1 at a MOI of 0.1 for 1, 4, 8, 12 h.p.i. and total RNA was extracted and analyzed for hsa-mir-101-1, hsa-mir-101-2 and miR-101 expression by quantitative real-time PCR. Data are normalized against uninfection and error bars present as means ± SD (n = 3). *P < 0.05. (**E**) Expression of hsa-mir-101-2 (n = 3) and miR-101 (n = 3) were detected by quantitative real-time PCR and correlation analysis showed that miR-101 was consistent with hsa-mir-101-2 (r^2^ = 0.5031, P < 0.05, linear regression).

Previous study reported that miR-101 was induced in the early stages of HSV-1 infection^22^. To optimize the time points, a time course of HSV-1 infection was performed to determine the suitable MOI for HSV-1 infection in HeLa cells. As show as in Fig. 1A, HeLa cells that were infected with HSV-1 at an MOI of 1 showed an almost 50% cytopathic effect (CPE) at 12 hours post-infection (h.p.i.); the MOI at which most cells were simultaneously infected without the generation of a typical CPE occurred at an MOI of either 0.1 or 0.01. Therefore, RNA was extracted from cells that were infected with HSV-1 at an MOI of 0.1 at 1, 4, 8 and 12 h.p.i. for miRNA expression analyses.

There are two loci of the miR-101 gene in the human genome. To determine whether HSV-1 induced the expression of both, unique primers were designed to amplify the precursors of mir-101-1 or mir-101-2. qRT-PCR showed that the level of the hsa-mir-101-1 precursor was mildly enhanced in HeLa cells at 4 and 8 h.p.i. and reached normal levels at 12 h.p.i. (Fig. 1B), whereas the expression of the hsa-mir-101-2 precursor was dramatically increased by 9 ~ 18-fold at 4, 8 and 12 h.p.i (Fig. 1C). This was consistent with mature miR-101 levels (Fig. 1D), and there was a positive linear correlation between the expression of the mir-101-2 precursor and mature miR-101 (Fig. 1E). These data indicate that the upregulation of miR-101 by HSV-1 infection may contribute to the activation of hsa-mir-101-2 precursor expression. Therefore, hsa-miR-101-2 induction by HSV-1 infection became the focus of further study.

### HSV-1 infection enhances miR-101-2/RCL1 promoter activity

**Figure 2:**
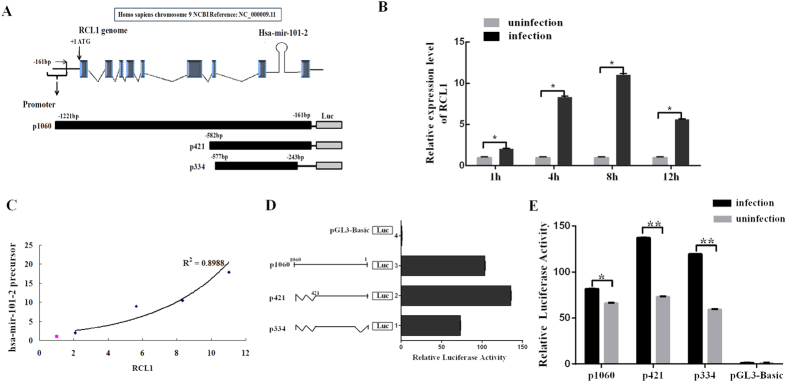
miR-101 coordinately expressed with host gene RCL1 and HSV-1 infection enhanced the activity of the corporate promoter. (**A**) The location of hsa-mir-101-2 on chromosome and the corporate promoter fragments. (**B**) HeLa cells were infected by HSV-1 at a MOI of 0.1 for 1, 4, 8, 12 h.p.i. and total RNA was extracted and analyzed for RCL1. Data are normalized against uninfection and error bars present as means ± SD (n = 3). *P < 0.05. (**C**) Correlation analysis showed that hsa-mir-101-2 was consistent with RCL (r^2^ = 0.8988, P < 0.05, linear regression). (**D**) HeLa cells were transfected with promoter fragments for 48 h and determined by luciferase activity assays. Data are normalized against the vector control and error bars present as means ± SD (n = 3). (**E**) HeLa cells were transfected with promoter fragments for 4 h followed by HSV-1 at a MOI of 0.01 for 8 h.p.i. Data are normalized against the vector control and error bars present as means ± SD (n = 3). *P < 0.05.

The hsa-mir-101-2 is located on chromosome 9: 4 850 291–4 850 381 within intron 8 of the RNA terminal phosphate cyclase-like 1 (RCL1) gene (Fig. 2A). In addition, HSV-1 infection induced the expression of RCL1 mRNA at an early stage (Fig. 2B). The expression of miR-101 and RCL1 was positively correlated in HSV-1 infection (Fig. 2C), suggesting that the RCL1 promoter drives the expression of both RCL1 and the hsa-mir-101-2 precursor. To characterize this promoter, different lengths of DNA fragments upstream of the RCL1 ATG codon were cloned into a pGL3-basic vector as the miR-101-2/RCL1 promoter (following plasmid names: p1060, p421 and p334) (Fig. 2A). A luciferase activity assay confirmed that all fragments had promoter activity, p421 was the most highly active (Fig. 2D). To determine whether HSV-1 infection affects the cloned promoter activity of miR-101-2/RCL1, HeLa cells were transfected with the plasmids and subsequently infected by HSV-1 at an MOI of 0.01. The result showed that Luciferase activity was significantly enhanced by HSV-1 infection at 8 h.p.i. (Fig. 2E). These results indicate that HSV-1 infection activates the miR-101-2/RCL1 promoter to enhance its expression.

### ICP4 binds to the miR-101-2/RCL1 promoter and induces its expression

**Figure 3:**
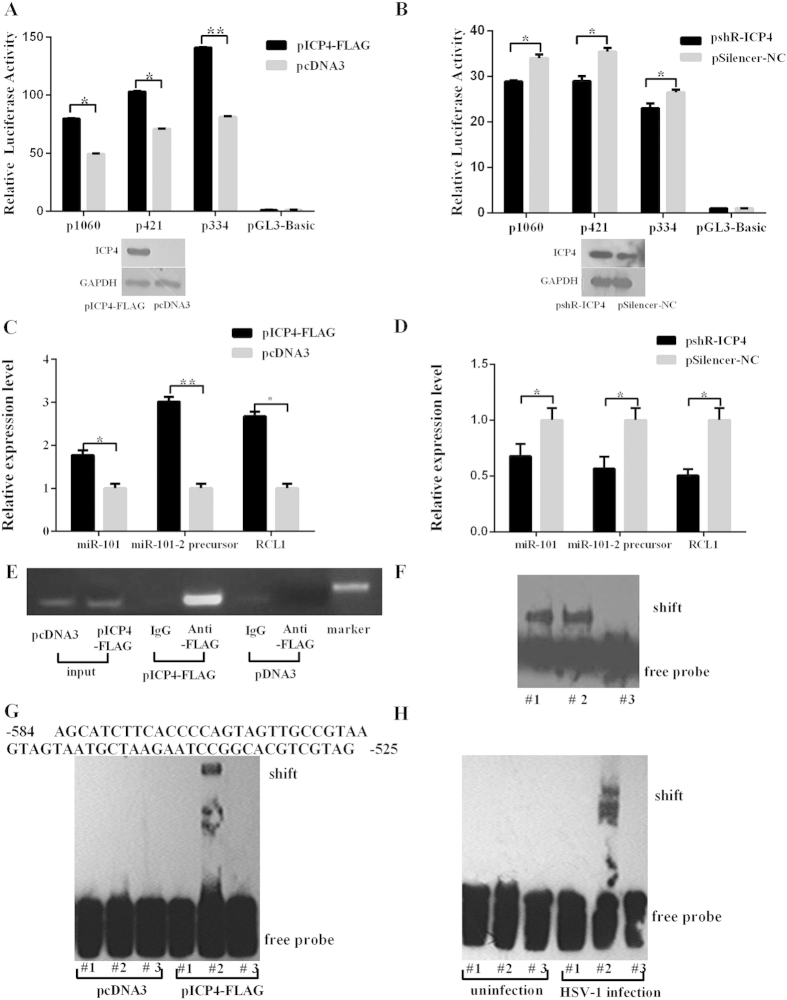
ICP4 mediates the expression of miR-101. (**A**) HeLa cells were co-transfected promoter fragments with pICP4-FLAG for 12 h or (**B**) co-transfected promoter fragments with pshR-ICP4 for 12 h followed by HSV-1 infection at an MOI of 0.01 for 8 h and determined by luciferase activity assays. The data are normalized to the vector control, and error bars are presented as the means ± SD (n = 3). *P < 0.05. ICP4 protein levels were detected by western blot transfected with ICP4-FLAG and pshR-ICP4. GAPDH protein was regarded as an endogenous normalizer. (**C**) HeLa cells were transfected with pICP4-FLAG for 12 h or (**D**) transfected pshR-ICP4 followed by HSV-1 infection at an MOI of 0.01 for 12 h. Total RNA was extracted and analyzed for hsa-mir-101-2 precursor, miR-101 and RCL1 expression using qRT-PCR. The data are normalized to uninfected controls, and error bars are presented as the means ± SD (n = 3). *P < 0.05. (**E**) ChIP primers are located at −347 bp ~ −586 bp upstream of the RCL1 gene and act as PCR primer controls. (**F**) The EMSA probes were PCR purified using the ChIP primers and were 3′ end-labeled by biotin then incubated with nuclear extracts from HeLa cells transfected with pICP4-FLAG, unlabeled competitor DNA at 50-fold molar excess. Lane #1 biotin-labeled probe incubated with 50-fold unlabeled probe and HeLa cell nuclear extracts, lane #2 biotin-labeled probe incubated with HeLa cell nuclear extracts, lane #3 biotin-labeled probe only. (**G**) Nuclear extracts of HeLa cells transfected with pICP4-FLAG incubated with the biotin-labeled probe (59 bp, 0.1 pmol), and unlabeled competitor DNA at a 200-fold molar excess. Lane #1 biotin-labeled probe incubated with 200-fold unlabeled probe and HeLa cell nuclear extracts, lane #2 biotin-labeled probe incubated with HeLa cell nuclear extracts, lane #3 biotin-labeled probe only. (**H**) Probe mobility was shifted by nuclear proteins from HeLa cells that were infected with HSV-1 at an MOI of 0.1 for 24 h. Lane #1 biotin-labeled probe incubated with 200-fold unlabeled probe and HeLa cell nuclear extracts, lane #2 biotin-labeled probe incubated with HeLa cell nuclear extracts, lane #3 biotin-labeled probe only.

ICP4 is encoded by an IE gene in the HSV-1 genome which functions as a transactivator^28^, and HSV-1 infection has been demonstrated to induce miR-101 expression. This prompted speculation as to whether ICP4 may mediate the upregulation of miR-101-2 expression during HSV-1 infection. To address this, ICP4 fused with a FLAG tag (pICP4-FLAG) was overexpressed and protein level were detected by western blot (Fig. 3A). A luciferase activity analysis was then performed in HeLa cells that were co-transfected with pICP4-FLAG and miR-101-2/RCL1 promoter vectors. After 12 h, ICP4 overexpression greatly enhanced luciferase activity compared to the control group (Fig. 3A). pshR-ICP4 was used to deplete ICP4 (Fig. 3B), and HeLa cells were co-transfected with the promoter vector and the pshR-ICP4; after 12 h, cells were infected with HSV-1 at an MOI of 0.01 for 8 h. The results indicate that luciferase activity induced by HSV-1 was reduced by 15% in the presence of pshR-ICP4 (Fig. 3B). This demonstrates that ICP4 can activate the miR-101-2/RCL1 promoter. Furthermore, to prove that ICP4 promotes the expression of miR-101 by enhancing its promoter activity, mRNA expression of RCL1 and hsa-mir-101-2 was assessed using ICP4 overexpression or knockdown in HeLa cells. The RNA expression levels of RCL1, the hsa-mir-101-2 precursor and mature miR-101 were found to be highly induced or reduced under ICP4 overexpression or knockdown conditions respectively (Fig. 3C,D). Taken together, these data indicate that ICP4 activates the miR-101-2/RCL1 promoter to induce the expression of miR-101 during HSV-1 infection.

To further demonstrate that ICP4 interacts with the miR-101-2/RCL1 promoter, a ChIP assay was performed using lysates from HeLa cells that overexpressed ICP4. The ChIP assay was performed using exogenous ICP4 fused with a FLAG tag, and an anti-FLAG antibody was used in this assay because the commercial anti-ICP4 antibody yielded nonspecific bands. PCR primers for amplifying fragments of the miR-101-2/RCL1 promoter from the ChIP complex were located −347 bp ~ −586 bp upstream of the miR-101-2/RCL1 promoter. As shown in Fig. 3E, there were strong PCR product bands when ICP4 was overexpressed, but these were not present in the IgG control or the negative control group and qPCR showed the same result (Fig. S1). This indicates that ICP4 can interact with the miR-101-2/RCL1 promoter.

To demonstrate that ICP4 directly binds to the miR-101-2/RCL1 promoter region and to validate the ChIP data, an EMSA was performed using a biotinylated probe. The EMSA probe DNA was purified from a PCR using the ChIP primers and was 3′ end-labeled by a biotin kit. The biotin probe was incubated with nuclear extracts from HeLa cells transfected with pICP4-FLAG, the constructs were separated by electrophoresis. A band shift resulted when the probe was incubated with ICP4 protein (Fig. 3F). Because the ICP4 binding sequence requires a tract of GC-rich sequences in the promoter^10^, the binding sequence was narrowed down to the region of −525 bp ~ −584 bp, and this fragment was artificially synthesized with a 3′ end biotin-labeled sequence to be used as a probe. EMSA was performed using this short probe, and the probe showed a shift following incubation of nuclear extracts from HeLa cells that had been transfected with pICP4-FLAG, but the control group had no such shift (Fig. 3G). This probe was then incubated with nuclear extracts from HeLa cells infected with HSV-1 at an MOI of 0.01 for 24 h. A band shift was observed in the infected group but not in the uninfected cells control group (Fig. 3H). A binding site (59 bp) deleted assay also performed, which resulted in loss of the promotor activity (Fig. S2). Together, the results indicate that HSV-1 infection induces the expression of miR-101 in an ICP4-dependent manner, and ICP4 directly binds to the miR-101-2/RCL1 promoter to activate its expression and the binding site region is required for promoter activity.

### GRSF1 is a target gene of miR-101

**Figure 4:**
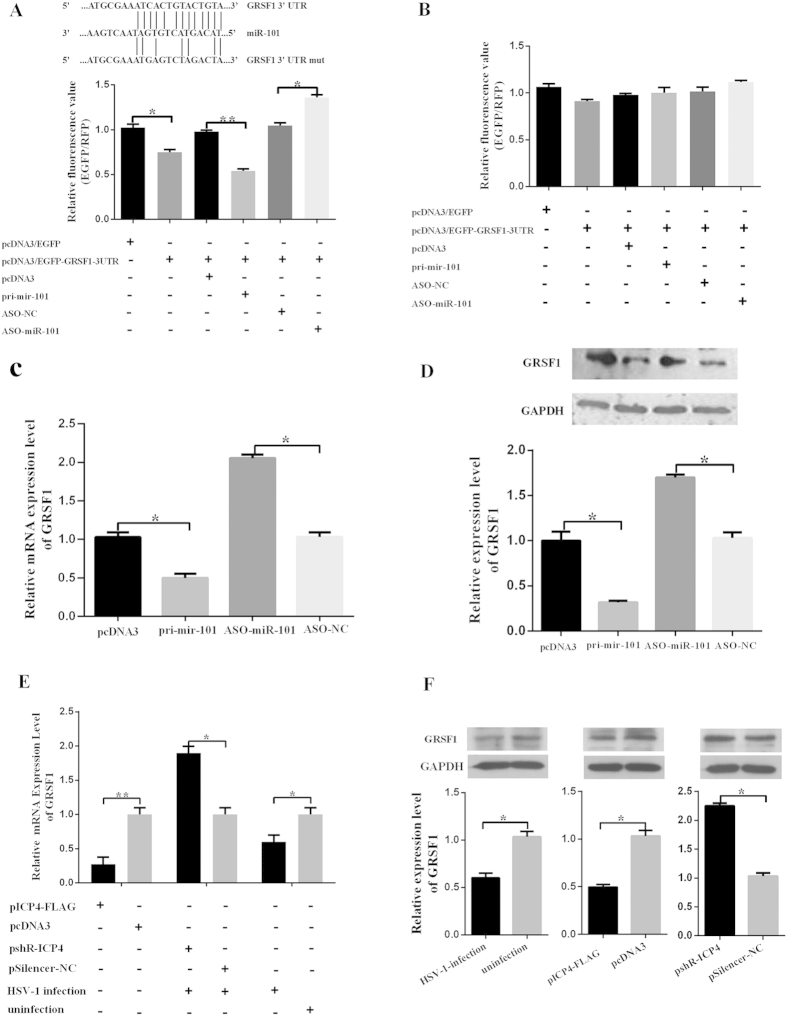
GRSF1 is a target gene of miR-101. (**A**) Top: TargetScan sequence predictions show that the GRSF1 3′UTR contains miR-101 binding sites. The GRSF1 3′UTR mutant contains a mutated miR-101 ‘seed region,’ and binding sites are shown. Bottom: HeLa cells were transfected with pcDNA3/EGFP, pcDNA3/EGFP-GRSF1-3′UTR, or pcDNA3/EGFP-GRSF1-3′UTR reporter vector along with pcDNA3/pri-miR-101, a control vector, miR-101-ASO or control ASO, as indicated. pDsRed2-N1 expressing RFP was also included for normalization. The fluorescence value in the control group was normalized to 1 (n = 3). *P < 0.05. (**B**) HeLa cells were transfected with pcDNA3/EGFP, pcDNA3/EGFP-GRSF1-3′UTR-mut or pcDNA3/EGFP-GRSF1-3′UTR-mut reporter vector along with pcDNA3/pri-miR-101, a control vector, miR-101-ASO or control ASO, as indicated. pDsRed2-N1 expressing RFP was also included for normalization. The fluorescence value in the control group was normalized to 1 (n = 3). *P < 0.05. (**C**) RNA was extracted from HeLa cells that had been transfected with pcDNA3/pri-miR-101, a control vector, miR-101-ASO or control ASO, and the GRSF1 mRNA level was measured by quantitative RT-PCR. Values were normalized to β-actin mRNA, and the level of GRSF1 mRNA in the control group was set to 1 (n = 3). *P < 0.05. (**D**) The GRSF1 protein level was measured by western blot of extracts from HeLa cells that have been transfected with pcDNA3/pri-miR-101, a control vector, miR-101-ASO or control ASO. GAPDH protein was used as an endogenous control. *P < 0.05. (**E**) RNA was extracted from HeLa cells that had been transfected with pICP4-FLAG and a control vector, transfected with pshR-ICP4 and a control vector followed by infection by HSV-1, only infected with HSV-1 or left as an uninfected control. The GRSF1 mRNA level was measured by qRT-PCR. Values were normalized to β-actin mRNA, (n = 3). *P < 0.05. (**F**) The GRSF1 protein level was measured by western blot of extracts from HeLa cells that had been transfected with pICP4-FLAG and a control vector, transfected with pshR-ICP4 and a control vector followed by infection by HSV-1, only infected with HSV-1 or left as an uninfected control. GAPDH protein was used as an endogenous control. *P < 0.05.

To elucidate the mechanism of miR-101 regulation of HSV-1 replication in host cells, it was necessary to identify the target genes of miR-101. The first step was to determine whether miR-101 directly targets HSV-1 genes or host cellular genes that are involved in the regulation of HSV-1 replication. Because no complementary sequences to the miR-101 seed sequence were found in HSV-1 transcripts by BLAST (http://blast.ncbi.nlm.nih.gov/Blast.cgi). TargetScan Release 6.0 was used to predict the cellular targets of miR-101. Based on the knowledge of gene function as pertains to antiviral activity, two gene were chosen among hundreds of candidate target genes: ATP synthase subunit beta (ATP5B) which was previously reported^22^ and GRSF1. In addition, GRSF1 was found to be involved in influenza virus infection^14^; thus, it was chosen for further study. To confirm whether miR-101 directly binds to the GRSF1 3′UTR and negatively regulates its gene expression, the GRSF1 3′UTR or a mutant 3′UTR (Fig. 4A) was cloned downstream of an EGFP gene in a pcDNA3 vector. HeLa cells were then transfected with the reporter vector along with the miR-101 expression vector or ASO-miR-101, which contains an antisense sequence of mature miR-101. The results show that the ectopic expression of miR-101 reduced the fluorescence intensity of EGFP, and ASO-miR-101 enhanced the expression of EGFP (Fig. 4A). However, when using a mutant vector containing a four-point mutation at the miR-101 seed sequence binding site, the fluorescence intensity of EGFP was not significantly changed by either overexpressing or inhibiting the expression of miR-101 (Fig. 4B). To further prove this relationship between miR-101 and GRSF1, qRT-PCR and western blot assays were used to determine the effect of miR-101 on endogenous GRSF1 expression. HeLa cells were transfected with pri-miR-101 or ASO-miR-101, and total RNA and protein were extracted after 48 h to assess endogenous GRSF1 expression. As shown in Fig. 4C,D, miR-101 overexpression was able to specifically reduce the level of endogenous GRSF1 mRNA by approximately 50% and protein by approximately 60% relative to the negative control. Nevertheless, blocking miR-101 expression resulted in a significant increase in GRSF1 mRNA and protein levels. The effects of HSV-1 infection and ICP4 on endogenous GRSF1 expression were then examined. The mRNA and protein expression levels of GRSF1 were significantly reduced under HSV-1 infection or ICP4 overexpression but were greatly increased under ICP4 knockdown conditions (Fig. 4E,F). These results indicate that miR-101 directly binds to the 3′UTR of GRSF1 mRNA, specifically reduces target gene expression, and plays an important role in the suppression of GRSF1 during HSV-1 infection.

### GRSF1 facilitates HSV-1 proliferation

**Figure 5:**
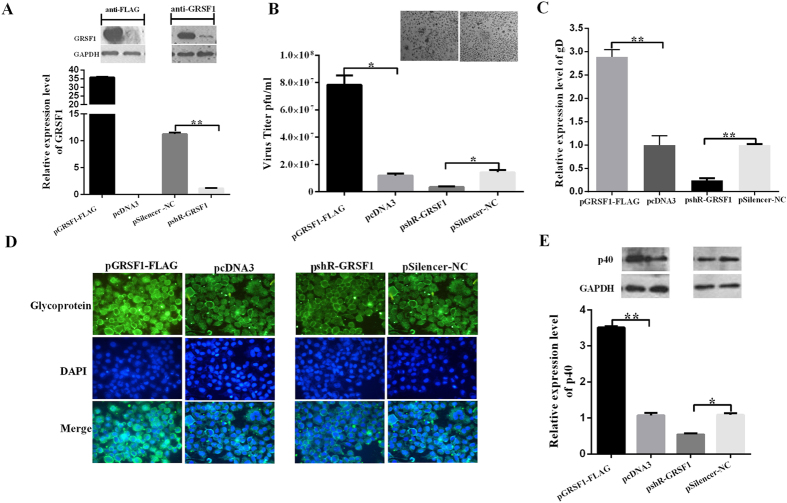
GRSF1 promotes HSV-1 replication. (**A**) GRSF1 protein levels were detected by western blot of extracts from HeLa cells that had been transfected with pGRSF1-FLAG and pshR-ICP4. GAPDH protein was used as an endogenous control. (**B**) HeLa cells were transfected with pGRSF1-FLAG, pshR-GRSF1 and their respective controls for 24 h followed by HSV-1 infection for 24 h. The supernatant was harvested at 24 h.p.i., and normal HeLa cells were infected. These data represent the mean values ± SD of at least three independent experiments. (**C**) The expression level of the gD gene was detected by qPCR under GRSF1 overexpression and knockdown conditions. These data represent the mean values ± SD of at least three independent experiments (n = 3). (**D**) Immunofluorescence was used to detect HSV-1 replication via the HSV-1 glycoprotein in HeLa cells under GRSF1 overexpression and knockdown conditions. (**E**) A western blot was used to detect HSV-1 replication in the presence of p40 under GRSF1 overexpression and knockdown conditions in HeLa cells. GAPDH protein was used as an endogenous control.

Because miR-101 directly targets the 3′UTR of GRSF1 and downregulates the expression of GRSF1, a GRSF1 expression plasmid (pGRSF1-FLAG) was generated that contained the open reading frame (ORF) of GRSF1 without the 3′UTR to avoid miR-101 interference. A GRSF1 siRNA expression vector (pshR-GRSF1) was also constructed (Fig. 5A). Several assays were then performed to investigate the role of GRSF1 in the regulation of HSV-1 replication. First, a plaque formation assay was applied to detect the effect of GRSF1 on HSV-1 replication. pGRSF1-FLAG, pshR-GRSF1 were transfected into cells and incubated for 24 h, followed by HSV-1 infection. After 24 h, the supernatant was harvested and then infected normal HeLa cells. When plaques formed, the number of plaques was counted. The results show that the overexpression of GRSF1 increased viral plaque formation by 4.5-fold, but a knockdown of GRSF1 repressed it by 60% (Fig. 5B). PCR techniques to measure DNA amplification are reported as sensitive methods for estimating the HSV-1 load in cells^29^. Real-time PCR was used with primers that targeted a sequence from the HSV-1 DNA gD gene for viral quantification. GRSF1 overexpression or knockdown was followed by HSV-1 infection at an MOI of 0.01, and the genome was extracted from whole cells and supernatant after 24 h. The result shows that GRSF1 overexpression increased the number of gD DNA copies by approximately 2.8-fold, whereas a GRSF1 knockdown reduced it by approximately 70% (Fig. 5C). Furthermore, the effect of GRSF1 on HSV-1 protein production was examined. Immunofluorescence and western blot assays showed that GRSF1 overexpression enhanced, but knockdown significantly decreased the expression levels of HSV-1 glycoprotein (Fig. 5D) and HSV-1 capsid protein p40 (Fig. 5E). Together, these results indicate that GRSF1, a target of miR-101, promotes HSV-1 proliferation.

### Restoration of GRSF1 expression counteracts the repression of miR-101 on HSV-1 proliferation

**Figure 6:**
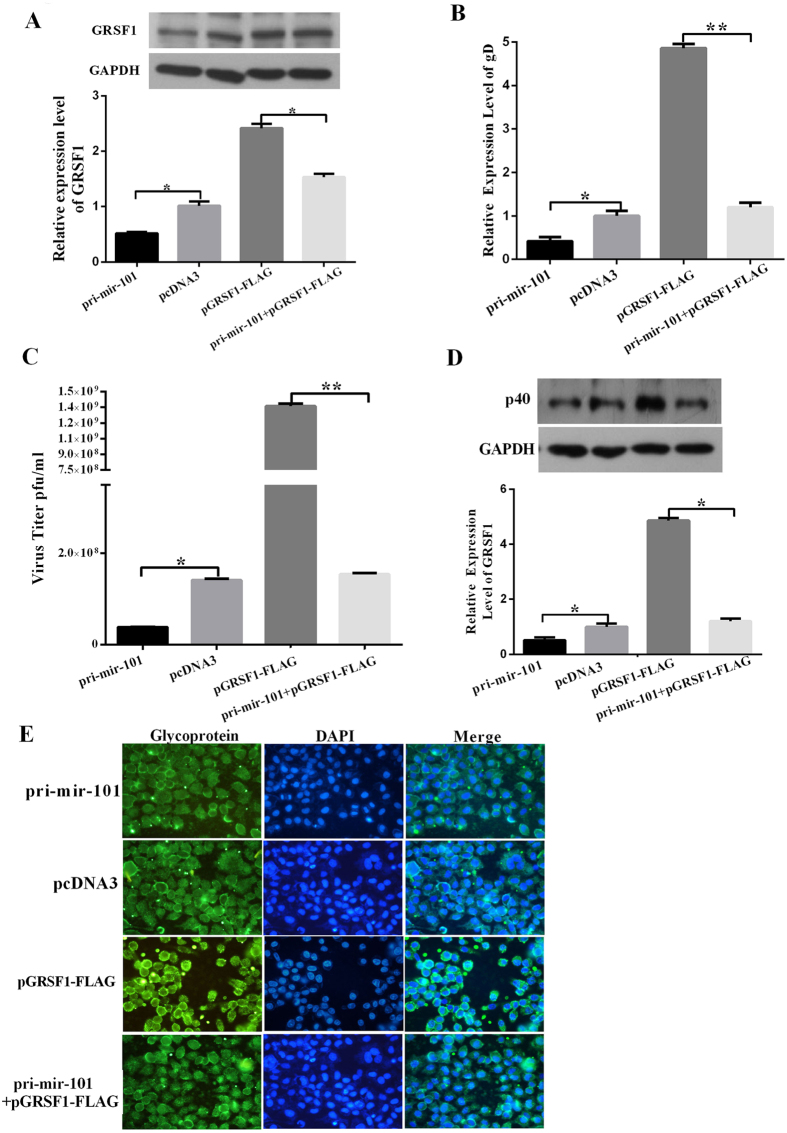
Overexpression of GRSF1 rescues the replication of HSV-1 that was repressed by pri-mir-101 in HeLa cells. (**A**) The overexpression of GRSF1 rescues the replication of HSV-1 that was repressed by pri-mir-101. HeLa cells were transfected with pcDNA3, pri-miR-101, or GRSF1-FLAG or were co-transfected with GRSF1-FLAG and pri-mir-101. GRSF1 protein levels were detected by Western blot. GAPDH protein was used as an endogenous control, and the relative quantity of GRSF1 protein is shown. (**B**–**D**) The overexpression of GRSF1 rescues the repression of HSV-1 replication that was caused by pri-mir-101. HeLa cells were transfected with pcDNA3, pri-miR-101, or GRSF1-FLAG or were co-transfected with GRSF1-FLAG and pri-mir-101. (**B**) The expression level of the gD gene was detected by qPCR. (**C**) Viral plaques in HeLa cells and (**D**) Western blot demonstrating HSV-1 replication in the presence of p40; GAPDH protein was used as an endogenous control. (**E**) Immunofluorescence was used to detect HSV-1 replication using an HSV-1 membrane protein in HeLa cells. *P < 0.05.

To confirm whether miR-101 regulates HSV-1 proliferation by targeting GRSF1, HeLa cells were first co-transfected with pri-mir-101-2 and pGRSF1-FLAG followed by HSV-1 infection at an MOI of 0.01 for 48 h. A series of experiments was then performed to analyze viral proliferation. As shown in Fig. 5, the reductions in GRSF1 protein levels (Fig. 6A), HSV-1 gD DNA copy numbers (Fig. 6B), viral plaques (Fig. 6C) and HSV-1 structural protein (glycoprotein and capsid protein p40) levels (Fig. 6D,E) that had been caused by miR-101 overexpression were reversed by the restoration of GRSF1 expression in HeLa cells. These results indicate that GRSF1 is a functional target of miR-101 that modulates HSV-1 proliferation in HeLa cells.

### The mechanism of GRSF1 promotes HSV-1 replication

**Figure 7:**
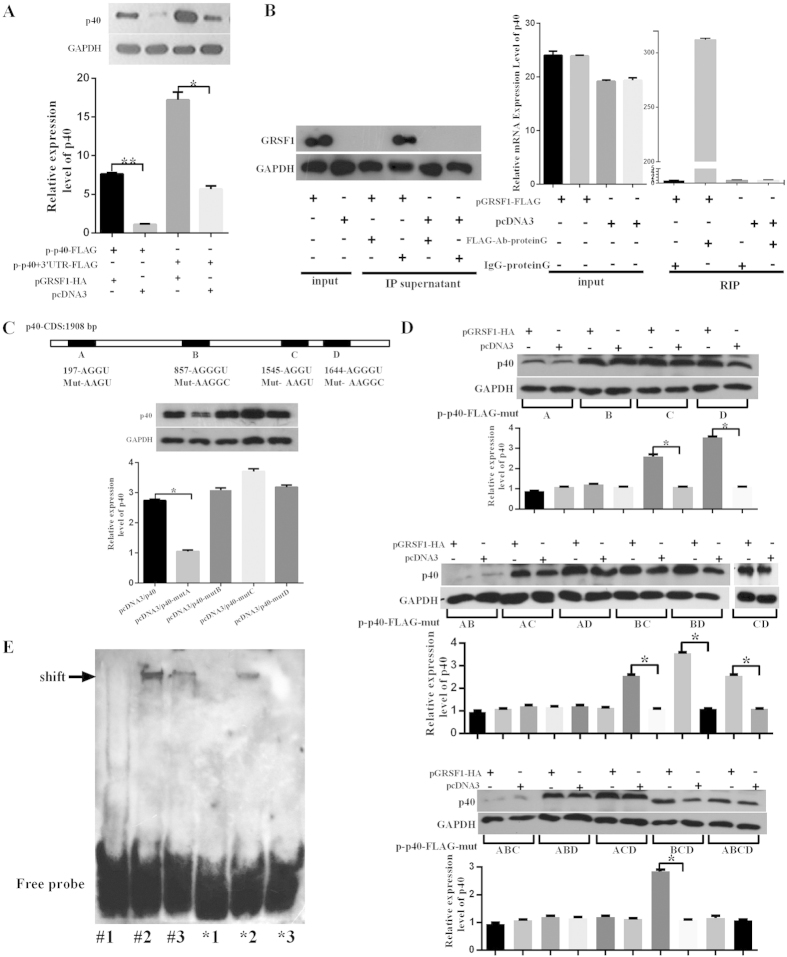
The mechanism of GRSF1 impacts HSV-1 replication. (**A**) HeLa cells were co-transfected with pGRSF1-HA and the p-p40-FLAG plasmid for 48 h to detect p40 by western blot, and GRSF1 induced the expression of p40 compared to the control group. GAPDH protein was used as an endogenous control. (**B**) HeLa cells were transfected with pGRSF1-FLAG and pcDNA3 for 24 h followed by HSV-1 infection for 24 h. The left column is an immunoprecipitation of GRSF1 using anti-FLAG and IgG from HeLa cells. The GRSF1 protein level in the supernatant after immunoprecipitation was detected by western blot. GAPDH protein was used as an endogenous control. The right column shows an immunoprecipitation of GRSF1 using anti-FLAG and IgG from HeLa cells, which allows for the identification of HSV-1 mRNAs. The mRNA level of p40 was detected by qRT-PCR. (**C**) Protein level of p40 was detected by western blot in extracts from HeLa cells that were transfected with pcDNA3/p40, pcDNA3/p40-mutA, pcDNA3/p40-mutB, pcDNA3/p40-mutC and pcDNA3/p40-mutD. GAPDH protein was used as an endogenous control. (**D**) The different mutations of predicted GRSF1 binding sites to p40 CDS and protein levels as detected by western blot in extracts from HeLa cells that had been transfected with mutant p40 plasmid and pGRSF1-HA expression vectors. GAPDH protein was used as an endogenous control. (**E**) Biotinylated RNA probes (6.25 nmol) for potential binding site A or the corresponding mutant site were incubated for 30 min at room temperature with 4 μg of recombinant GRSF1 protein and resolved on a 6% polyacrylamide gel. The arrow indicates the band-shifted complexes. For the competition assay, unlabeled competitor RNA at 50-fold molar excess was added. Lane #1 is biotin-labeled p40 probe only, lane #2 is biotin-labeled p40 probe incubated with GRSF1 protein and lane #3 is biotin-labeled probe incubated with 50-fold unlabeled p40 probe and GRSF1 protein. Lane *1 is biotin-labeled p40-mut probe, lane *2 is biotin-labeled p40-mut probe incubated with GRSF1 protein and lane *3 is biotin-labeled p40-mut probe incubated with 50-fold unlabeled p40-mut probe and GRSF1 protein.

GRSF1 protein binds to the 5′UTR of the influenza virus nucleocapsid gene transcript and promotes the translation of viral mRNAs to facilitate viral replication in infected cells^14^,^30^. To determine whether GRSF1 binds to HSV-1 gene transcripts to enhance their translation, the HSV-1 p40 gene was analyzed. HSV-1 p40 expression vectors that contained the p40 ORF (coding sequence, CDS) only [pcDNA3/FLAG-p40-CDS (p-p40-FLAG)] or both the p40 ORF and the whole 3′UTR with 56 nucleotides [pcDNA3/FLAG-p40-CDS-3′UTR (p-p40 + 3′UTR-FLAG)] were generated. GRSF1 and the p40 expression vectors were then co-transfected into HeLa cells, and the level of p40 protein was detected using an anti-p40 antibody. The results showed that GRSF1 significantly enhanced the expression of p40 with both the p-p40-FLAG and p-p40 + 3′UTR-FLAG constructs by approximately 7.6-fold and 3.5-fold, respectively (Fig. 7A), suggesting that GRSF1 facilitates the expression of HSV-1 in a p40-3′UTR-independent manner. A RIP assay was then performed to confirm the interaction between the GRSF1 protein and p40 mRNA. HeLa cells were transfected with GRSF1-FLAG and infected by HSV-1. Then, GRSF1 and any associated RNA were immunoprecipitated using an anti-FLAG antibody, and qRT-PCR was used to detect p40 mRNA. The results showed that the FLAG antibody efficiently pulled down GRSF1, and the p40 mRNA that bound to GRSF1 was abundantly enriched (Fig. 7B). A sequence analysis of GRSF1 interacting with the influenza virus 5′UTR showed that GRSF1 binds to the consensus sequence motifs AGGU and AGGGU^14^. Accordingly, an analysis of the p40 mRNA demonstrated that there are four positions that include the sequence AGGU/AGGGU in the p40 CDS, but not in the 3′UTR, which explains why GRSF1 had the same effect on the p40-CDS and p40-CDS-3′UTR. Therefore, these four potential binding sites were mutated without changing the peptide sequence (Fig. 7C). Plasmids that overexpressed wild type (WT) p40 and mutant p40 were transfected into HeLa cells, and western blots were used to detect the protein levels. As shown Fig. 6C, p40 mutants B, C and D showed the same level of expression as wild type p40, but p40 mutant A demonstrated a significantly reduced level of protein. In addition, different combination mutants were generated for the four potential sites and were co-transfected with pGRSF1-HA. The results showed that all p40 mutants that contained the site A mutant and were co-transfected with pGRSF1-HA had a similar level of p40 expression when compared to the control groups, while the wild type site A p40 plasmid co-transfected with pGRSF1-HA showed a higher level of p40 expression when compared to the control groups. These results indicate that endogenous GRSF1 may bind to site A and promote the expression of p40, but the facilitation caused by GRSF1 was abolished when site A was mutated. RNA EMSA experiments were then carried out using biotinylated wild type or mutated p40 site A mRNA fragments as probes to confirm a direct interaction between p40 mRNA and GRSF1 protein (Fig. 7D). The RNA biotin probe was pre-incubated with GRSF1 protein extract from HeLa cells. The results showed that the GRSF1 protein formed complexes with the wild type p40 RNA probe, but the mutant probes were much less efficient (Fig. 7E). The binding specificity was confirmed by the displacement of the bound fraction with an excess of unlabeled RNA probe. These results demonstrate that GRSF1 protein directly binds to p40 mRNA to enhance its translation.

## Discussion

**Figure 8:**
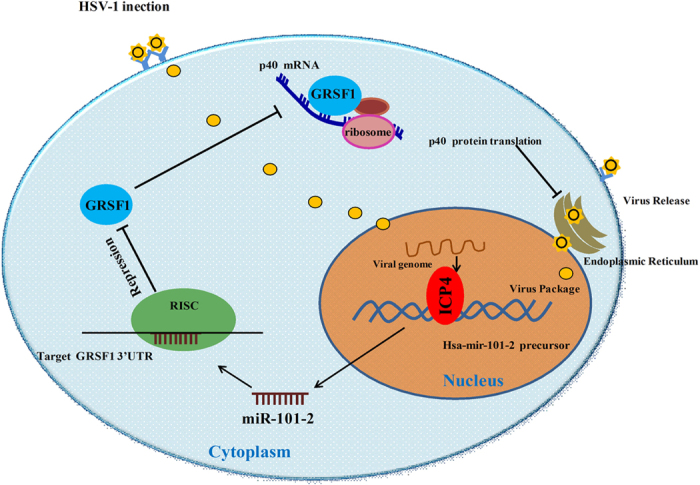
Model of ICP4-induced miR-101 regulates HSV-1 replication. The upregulation of ICP4-induced miR-101 during HSV-1 infection decreases GRSF1 expression. ICP4 directly binds to the hsa-mir-101-2 promoter and acts as a transactivator. Consequently, miR-101 represses the expression of GRSF1 via base pairing between miR-101 and the 3′UTR of GRSF1. The repression of GRSF1 leads to the reduction of p40 translation, which is usually enhanced by GRSF1 binding to p40 mRNA, and the promotion of its recruitment to ribosomes to decrease the production of viral progeny.

The life cycle of HSV-1 begins when cells are first invaded by HSV-1, at which point lytic and replicative infection begins and many more viral particles are produced, leading to cell death then the virions released to the surrounding cells or tissues, and they switch to latent infection mode^31^. These two processes of HSV-1 infection are complex. One potential problem is that the virus can lead to lytic host cell death due to the presence of viral particles. Additionally, viral infection will inevitably cause a change in host cellular protein or RNA expression levels, which can result in lytic host cell death. By monitoring and manipulating the host cellular environment, the virus must create conditions that are suitable for appropriate re-infection by maintaining the survival of the host cells, as the death of host cells may lead to the loss of the latent viral genome. Several studies have explored that viral infection induces or inhibits cellular miRNAs which contribute to the promotion or defense of virus and host cell proliferation. Our previous studies have suggested that miR-101 is significantly induced by HSV-1 infection at an early stage. Here we fist optimize the time points of infection and virus titer. Our previous experiments showed that HeLa cells infected using high MOI and for long time appeared CPE of the majority of cells, which may result in different stages (early and late stage) for infected cells, thus we used low MOI to infect cells in short time (early stage) to obtain reliable result of inducing miR-101 expression. So this procedure may avoid paracrine effects of HSV-1 infection because that HSV-1 usually releases offspring in cells at 18 h post-infection^32^. This study describes a model in which the expression of hsa-mir-101-2 is induced during HSV-1 infection in HeLa cells (Fig. 8). There are two loci of the miR-101 gene in the human genome: hsa-mir-101-1 and hsa-mir-101-2 which are located on chromosome 1 and chromosome 9, respectively. We first elucidate that HSV-1 mainly induced miR-101-2 in infection (Fig. 1C). Because the expression of miR-101 is induced at an early stage of HSV-1 infection, ICP4 of HSV-1 which is a transactivator of the promoter required for the expression of early and late genes of HSV-1^6^,^7^,^33^,^34^ was considered to be involved in this process. Overexpressing of ICP4 enhances the luciferase activity of the miR-101-2/RCL1 promoter and elevates the level of miR-101 expression, but depletion of ICP4 represses (Fig. 3). These data indicate that ICP4 activates miR-101 expression.

Furthemore, ICP4 localizes to viral replication compartments in the nuclei of infected cells^35^ and act as a transcriptional activator and repressor^36^,^37^, and broadly activates different promoters^34^. To confirm whether ICP4 directly regulates hsa-mir-101-2 or indirectly regulates the promoter by affecting cellular factors to enhance the expression of miR-101, a ChIP assay and EMSA were performed. The results showed that ICP4 can directly bind to the miR-101-2/RCL1 promoter and act as a transactivator (Fig. 3), and identified that ICP4 bindng site in the miR-101-2/RCL1 promoter is approximately 50 nt fragment. This long sequence segment may be related to the long and narrow structural characteristics of ICP4. A recent study reported that the expression of VEGF-A was directly induced by ICP4, and ICP4 binds to the VEGF-A promoter sequence^10^, and found that ICP4 binds the GC-rich sequences in the VEGF-A promoter, not ICP4 DNA binding consensus sequence of A/GTCGTCNNNNYCGRC (N = any nucleotide, Y = pyrimidine, R = purine)^38^,^39^. Similarly, our study demonstrated that ICP4 acts as a transactivator and directly binds to the GC-rich sequence of miR-101-2/RCL1 promoter region which is similar with the VEGF-A promoter. Because ICP4 may go through extensive post-translational modifications that could alter sequence affinity, ICP4 may bind to a variety of sequences with no apparent relation to its consensus sequence^5^,^40^. However, both specific and nonspecific DNA binding sequences are crucial for ICP4 function. In addition, nonspecific ICP4 binding sites have been confirmed to be associated with transcriptional activation^41^,^42^. The present study examining the function of ICP4 as it relates to miR-101-2/RCL1 promoter activity identified the binding site between ICP4 and the miR/RCL1-101-2 promoter sequences as a nonspecific sequence (Fig. 3G), which confirms the transcriptional activation of the miR-101-2/RCL1 promoter by ICP4 by an alternate method. These results indicate that the HSV-1 induction of miR-101 expression immediately depends upon ICP4, which is a key HSV-1 molecule, aside from other cellular transcription activators that are affected by HSV-1 infection. Therefore, ICP4 could be engineered to directly bind to the miR-101-2/RCL1 promoter region and induce the expression of miR-101.

Induced miR-101 expression is dependent on ICP4, the HSV-1 transactivator, and the HSV-1 transcriptional regulation program drives miR-101 expression. In addition, miR-101 mediates a number of innate immune response factors that may be beneficial to the pathogen. The mechanisms that promote persistent HSV-1 infection have remained largely obscure. This process likely involves a lytic growth phase, including replication of the viral genome, the production of progeny virus, the lysis of host cells, and the infection of neighboring cells. This will lead to a drastically reduced expression of most viral genes and the production of infectious progeny that can evade immune responses to ensure host cell survival^43^. In the search for the miR-101 target gene, two genes were discovered that were involved in the process of viral infection: ATP5B^22^ and GRSF1, which promotes HSV-1 replication (Fig. 5). This study showed that GRSF-1 promotes the replication of HSV-1 by binding to HSV-1 mRNA to facilitate its translation (Fig. 5). GRSF1 has been implicated in influenza infections as a positive-acting translational regulatory factor by binding to the 5′UTRs of influenza RNAs. This study indicated that GRSF1 enhances HSV-1 replication by inducing p40 translation. A recent report also suggested that GRSF-1 binds to the mRNA of the SNARE protein Use1 and possibly positions the 40S ribosomal subunit and associated translation factors upstream of the translation start site to stimulate Use1 translation^44^. It also interacts with eukaryotic initiation factors to efficiently recruit ribosomes to viral mRNAs to stimulate viral mRNA translation^30^. The related report also demonstrated that GRSF1 interacts with the specific sequences AGGU and AGGGU within the influenza viral 5′UTR to selectively promote viral mRNA translation and virus infection. The present study found that GRSF1 promotes the expression of p40, and a sequence analysis of p40 mRNA uncovered four positions containing the AGGU/AGGGU sequence (Fig. 7C). The RIP assay, mutant binding sites assay and RNA EMSA results showed that GRSF1 directly binds to p40 mRNA and promotes the translation of p40 (Fig. 7B). The RNA EMSA showed that, when the mutant binding site probe is incubated with the GRSF1 protein, the shift is less evident than with the wild type probe (Fig. 7E), but the binding interaction does not disappear. A possible explanation is that the site was mutated from AGGU to AAGU, with a change in just one nucleotide, and the 3′ nucleotides G and U remained unchanged to avoid altering the sequence of amino acids. In the AGGU/AGGGU consensus GRSF1 binding site, the 3′ nucleotides G and U were shown to be important determinants for GRSF1 binding and for the recruitment of viral mRNAs to ribosomes^14^; thus, the mutant probe can also form a complex with GRSF1. The mechanism of p40 upregulation induced by GRSF1 to enhance HSV-1 replication may be the same as that of the GRSF1 promotion of influenza virus infection, which means that GRSF1 enhances p40 translation by binding to p40 mRNA and promoting its recruitment to ribosomes so as to improve the production of viral progeny.

In summary, we have identified a molecular mechanism by which host miRNA and HSV-1 cooperate to establish an environment that provides HSV-1 replication (Fig. 8). HSV-1 infection induces the expression of miR-101 as a result of ICP4 binding to the promoter region of miR-101-2/RCL1, which promotes its transcription. GRSF1 is a target gene of miR-101 and is repressed by miR-101, and GRSF1 promotes HSV-1 replication by upregulating p40 to enhance HSV-1 replication. Meanwhile, miR-101 represses the expression of GRSF1 to decrease HSV-1 replication to ensure the survival of host cells, thus supporting persistent HSV-1 infection. These findings gain insights on mechanism underlying interactions of host cells and HSV-1 and might be potential value for the development of antiviral therapy.

## Experimental Procedures

### Cell culture and transfection

HeLa cell lines (ATCC, USA) were propagated and maintained in RPMI 1640 medium (Invitrogen, Carlsbad, CA) supplemented with 10% fetal bovine serum and 1% antibiotics, in a humidified atmosphere at 37 °C with 5% CO2. Transfected reaction is incubated in antibiotic-free Opti-MEM medium (Invitrogen, Carlsbad, CA) with lipofectamine 2000 reagent (Invitrogen, Carlsbad, CA).

### Virus infection

HSV-1 Stocker strain was propagated on HeLa cells. At the peak of the cytopathogenic effect, viruses were harvested by three cycles of freezing and thawing. After centrifugation, the supernatant was titrated by plaque assays, and stored at –80°C. HeLa cells were infected with HSV-1 at MOI of 0.1 or 0.01 for 24 h.p.i. HSV-1 yields were determined by standard plaque assays after 2-day incubation.

### Plasmid construction

The primary miR-101 was amplified from genomic DNA and cloned into the pcDNA3 vector sites (*BamHI* and *EcoRI*). We aslo commercially synthesized the 2′-O-methylmodified antisense oligonucleotides of miR-101 (ASO-miR-101) as the inhibitor of miR-101. The 3′UTR of GRSF1 containing the miR-101 binding site and the mutant 3′UTR fragment with mutant binding site of miR-101 were obtained by annealing double-strand DNA and inserted into the pcDNA3/EGFP vector with the *BamHI* and *EcoRI* sites. Thus the GRSF1 3′UTR or mutant 3′UTR was inserted into the downstream of EGFP and the resulting plasmids were used as reporter vectors.

The pSilencer/shR-GRSF1 and ICP4 plasmid expressing a siRNA targeting *GRSF1* and *ICP4* transcript was constructed by annealing single-strand hairpin cDNA and inserting it into a pSilencer2.1-U6 neo vector (Ambion, Austin, TX, USA) using *BamHI* and *HindIII* sites. The full-length sequence of human *GRSF1* and *ICP4* cDNA deposited in Genbank was cloned into *EcoRI/Xhol* restriction sites of pcDNA3. The resulted plasmids were termed as pGRSF1 and pICP4.

The promoter of miR-101 was amplified from genomic DNA and cloned into *KpnI/EcoRI* restriction sites of pGL3-Basic (Promega) upstream of the firefly luciferase gene. All of the primers for PCR amplification are illustrated in Table S1.

### Luciferase assay

Luciferase activity of promoter was evaluated by Dual-Luciferase Reporter Assay System (Promega). HeLa cells were transfected with p1060/p421/p334-pGL3-Basic or co-transfected with pICP4-FLAG and pshR-ICP4 plasmids. Total protein was preparated after 8 h post-transfection. The 65 μl of each sample extract was used to detect luciferase activity. pRL-TK vector as an internal control.

### Chromatin immunoprecipitation assay

*In vivo* binding of ICP4 to the promoter of has-mir-101-2 was investigated using the ChIP assay according to the protocol of EpiQuik^TM^ Chromatin Immunoprecipitation Kit (Epigentek Group Inc, USA). Briefly, HeLa cells were transfected with pICP4-FLAG. After 24 h, cells were trypsinized and collected, after washed by PBS followed by treated with 1% formaldehyde for 10 min at 37 °C and washed twice in ice-cold PBS. Samples were sonicated to shear DNA to lengths between 200 and 1000 bp. Subsequently, an aliquot of sheared DNA was analyzed by agarose gel electrophoresis to confirm sheared DNA length and standardize protein-DNA complex input for immunoprecipitation. The chromatin samples were incubated overnight at room temprature with mouse anti-FLAG monoclonal antibody (MBL, Japan). DNAs were purified by EpiQuik^TM^ Chromatin Immunoprecipitation Kit. PCR was performed with primers flanking the predicted ICP4 binding site in the promoter sequence. All of the primers for PCR amplification are illustrated in Table S1.

### Electrophoretic mobility shift assay

Nuclear proteins from HeLa cells transfected with pICP4-FLAG and infected by HSV-1 were extracted using 0.3 M sucrose, 60 mM NaCl, 15 mM Tris-Cl, 10 mM EDTA. The probe between −347 bp ~ −586 bp was biotin-labeled using Biotin 3′ End DNA Labeling Kit (Thermo, Pierce, USA), and the probe between −525 bp and −584 bp was artificially synthesized with 3′ end biotin labeled from GENEWIZ (Beijing, China). Binding assays were performed in 20 μl of reaction mixture system containing 2 μg of nuclear proteins, 5 mM MgCl, 2.5% Glycerol, 0.05% NP-40, 1 ng/μl of poly (dI·dC) and 4 pmol labeled probes at 37 °C for 20 min. Reactions were analyzed by electrophoresis on a 6.0% non-denaturing polyacrylamide gel at 100 V. After transfer, the membrane was immediately cross-linked for 5 min by UV-light and bands detected by chemiluminescence (Thermo, Pierce, USA). All of the probe sequence is illustrated in Table S1.

### Fluorescent report assays

HeLa cells were transfected in 48-well plates with pcDNA3/pri-miR-101, control vector, ASO-miR-101, or ASO-NC, and then with the reporter vector pcDNA3/EGFP-GRSF1-UTR or pcDNA3/EGFP-GRSF1-mut-UTR the following day. The vector pDsRed2-N1 (Clontech, Mountain View, CA), expressing RFP (red fluorescent protein), was spiked in and used for normalization. 48 h later, the cells were lysed with RIPA lysis buffer (0.1%SDS, 1%NP-40, 1 mM MgCl, 10 mM Tris-Cl, pH 8.3). The intensities of EGFP and RFP fluorescence were detected with a Fluorescence Spectrophotometer F-4500 (Hitachi, Tokyo, Japan).

### DNA extraction

DNA was extracted from HSV-1 infected HeLa cells. 500 μl of lysis buffer (10 mM Tris-HCl pH 7.4, 0.1 mM EDTA, 0.5% sodium dodecyl sulfate, 20 μg/ml RNase A) was added to cells and incubated for 1 h at 37 °C. Proteinase K was added to cell lysate at 100 μg/ml and incubated overnight at 50 °C. The samples were then extracted twice with phenol-chloroform-isoamyl alcohol (25/24/1, vol/vol). The DNA was precipitated with anhydrous ethyl alcohol in the presence of 0.3 M sodium acetate. The DNA pellets were washed with 70% alcohol, dried, and suspended in 100 μl of TE buffer. The DNA preparations were stored at −20 °C.

### Quantitative real-time PCR

A cDNA library was generated through reverse transcription using M-MLV reverse transcriptase (Promega) with 2 μg of RNA extracted from the HeLa cells. The cDNA was used for the amplification of *RCL1*, *GRSF1* and *β-actin* gene was used as an endogenous control for the PCR reaction. For the detection of mature miR-101, hsa-mir-101-2, hsa-mir-101-1, with U6 snRNA as endogenous control, 2 μg of small RNA was reverse transcribed to cDNA using M-MLV reverse transcriptase (Promega). The relative level of HSV-1 DNA was detected by *gD* gene. 18S rRNA was used as the internal control gene^45^. The PCR reaction was performed with the corresponding PCR primers, which ensured the specificity of the PCR products. PCR cycles were as follows: 94 °C for 4 min, followed by 40 cycles of 94 °C for 30 s, 58 °C for 30 s, and 72 °C for 30 s. Ultra SYBR Mixture (CWBIO, Beijing, China) was used following the manufacturer’s instructions, and real-time PCR was performed and analyzed by the iQ5 Real-Time PCR Detection system (Bio-Rad, CA, USA). All of the primers for PCR amplification are illustrated in Table S1.

### Western blot

Total proteins from transfected HeLa cells were extracted 48 h post-transfection using RIPA, and protein expression was analyzed by western blot. GAPDH served as a loading control. The following antibodies were used: mouse anti-GRSF1, mouse anti-FLAG, rabbit anti-GAPDH, and goat anti-rabbit, goat anti-mouse (Tianjin Saier Biotech, China). Monoclonal antibody gainst nucleocapsid (p40) of HSV-1 antibody was generated by BALB/c mice lymphocyte hybridom HB-8068 (ATCC, USA). Bands quantified with Labworks 4.0 software.

### HSV-1 viral plaque assay

HeLa cells were seeded in the 48-well plates at a density of 2 × 10^4^ in the normal medium 18–24 hours before HSV-1 infection then infected with serial dilutions of HSV-1 containing supernatants by incubation for 1.5 h at 37 °C with 5% CO2. After 24 h, the supernatant was harvested and then infected normal HeLa cells by incubation for 1.5 h and replace the medium by the plaquing medium prepared by mixing a sterile solution of 0.8% methyl cellulose in H_2_O and 1% FBS containing 1640 and incubated at 37 °C with 5% CO2 until the plaques became visible, the viral plaques were counted.

### Immunofluorescent staining

HeLa cells were transfected with plasmid in 14-well. After 48 h post-transfection, cells were fixed for 30 min in 4% paraformaldehyde in phosphate-buffered saline followed by permeabilization with 0.05% Triton-X-100 for 5 minutes then blocked with 10% donkey serum albumin and incubated with the primary HSV-1-glycoprotein antibody (Saier Biotech, Tianjin, China) followed by secondary FITC-conjugated anti-mouse IgG or FITC-conjugated anti-Rabbit IgG. Cell nuclei were also stained with diamidino-2-phenylindole hydrochloride (DAPI) for 5 min. Immunofluorescent samples were examined under laser scanning microscope.

### RNA immunoprecipitation

RNA immunoprecipitation was performed mainly as described in^46^; lysis buffer II (10 mM HEPES pH 7.0, 100 mM KCl, 5 mM MgCl2, 25 mM EDTA, 0.5% Nonidet P-40, 1% Triton X-100, 0.1% sodium dodecyl sulfate, 10% glycerol) was used to prepare HeLa total extracts prior to immunoprecipitation.

### RNA electrophoretic mobility shift assay

Proteins extracted from HeLa cells were transfected with pGRSF1-FLAG. The probe was artificially synthesized with 3′ end-labeled biotin from GenePharma (Shanghai, China). Binding assays were performed in 20 μl of reaction mixture system containing 2 μg of GRSF1 protein, tRNA (10 mg/mL) 0.2 ug, 5% Glycerol and 4 pmol labeled probes at 37 °C for 20 min. Reactions were analyzed by electrophoresis and bands detected by chemiluminescence (Thermo, Pierce, USA). All of the probe sequence is illustrated in Table S1.

### Statistics and data analysis

Statistical significance was determined using the Student’s t test. In all figures, values are expressed as mean ± standard deviation (SD), and statistical significance (P < 0.05) is indicated by a single asterisk. The data generated *in vitro* are representative of at least three separate experiments conducted in triplicate.

## Additional Information

**How to cite this article**: Wang, X. *et al.* ICP4-induced miR-101 attenuates HSV-1 replication. *Sci. Rep.* **6**, 23205; doi: 10.1038/srep23205 (2016).

## Supplementary Material

Supplementary Information
