# Author Correction: ICP4-induced miR-101 attenuates HSV-1 replication

**DOI:** 10.1038/s41598-021-95585-4

**Published:** 2021-08-06

**Authors:** Xiangling Wang, Caifeng Diao, Xi Yang, Zhen Yang, Min Liu, Xin Li, Hua Tang

**Affiliations:** Tianjin Life Science Research Center and Department of Pathogen Biology, School of Basic Medical Sciences, Tianjin Medical University, 22 Qi-Xiang-Tai Road, Tianjin, 300070 China

Correction to: *Scientific Reports* 10.1038/srep23205, published online 17 March 2016

This Article contains errors in Figures 5 and 6.

In panel D of Figure 5, the images Glycoprotein and Merge for pSilencer-NC are a duplication of the images Glycoprotein and Merge for pcDNA3. The correct Figure 5 appears below.

**Figure 5:**
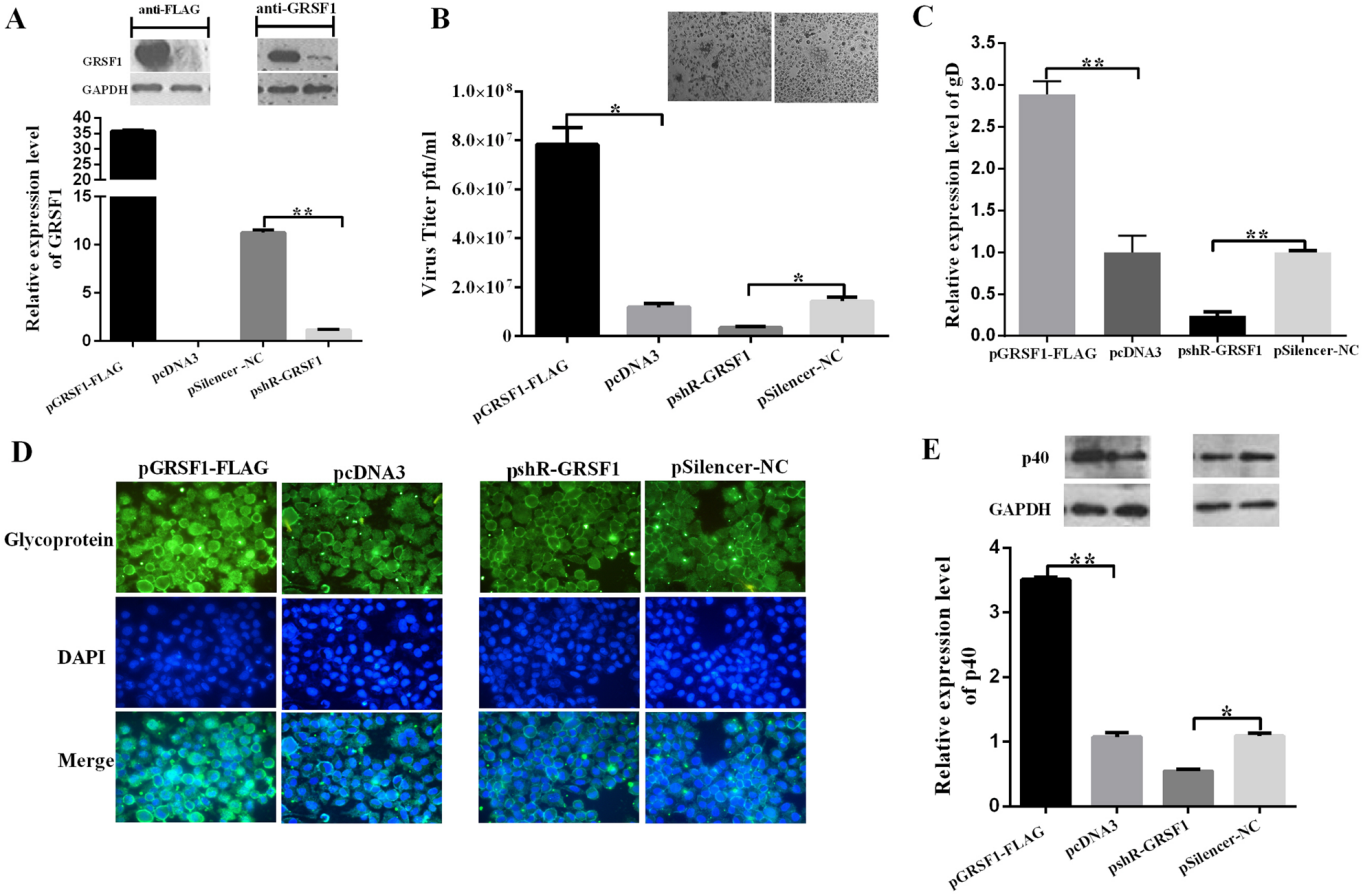
GRSF1 promotes HSV-1 replication. (**A**) GRSF1 protein levels were detected by western blot of extracts from HeLa cells that had been transfected with pGRSF1-FLAG and pshR-ICP4. GAPDH protein was used as an endogenous control. (**B**) HeLa cells were transfected with pGRSF1-FLAG, pshR-GRSF1 and their respective controls for 24 h followed by HSV-1 infection for 24 h. The supernatant was harvested at 24 h.p.i., and normal HeLa cells were infected. These data represent the mean values ± SD of at least three independent experiments. (**C**) The expression level of the gD gene was detected by qPCR under GRSF1 overexpression and knockdown conditions. These data represent the mean values ± SD of at least three independent experiments (n = 3). (**D**) Immunofluorescence was used to detect HSV-1 replication via the HSV-1 glycoprotein in HeLa cells under GRSF1 overexpression and knockdown conditions. (**E**) A western blot was used to detect HSV-1 replication in the presence of p40 under GRSF1 overexpression and knockdown conditions in HeLa cells. GAPDH protein was used as an endogenous control.

In panel E of Figure 6, all images for pcDNA3 are a duplication of the corresponding pcDNA3 images in panel D of Figure 5. Additionally, the image DAPI for pri-mir-101 + pGRSF1-FLAG is a duplication of the image DAPI for pcDNA3 in panel D of Figure 5. The correct Figure 6 appears below.

**Figure 6:**
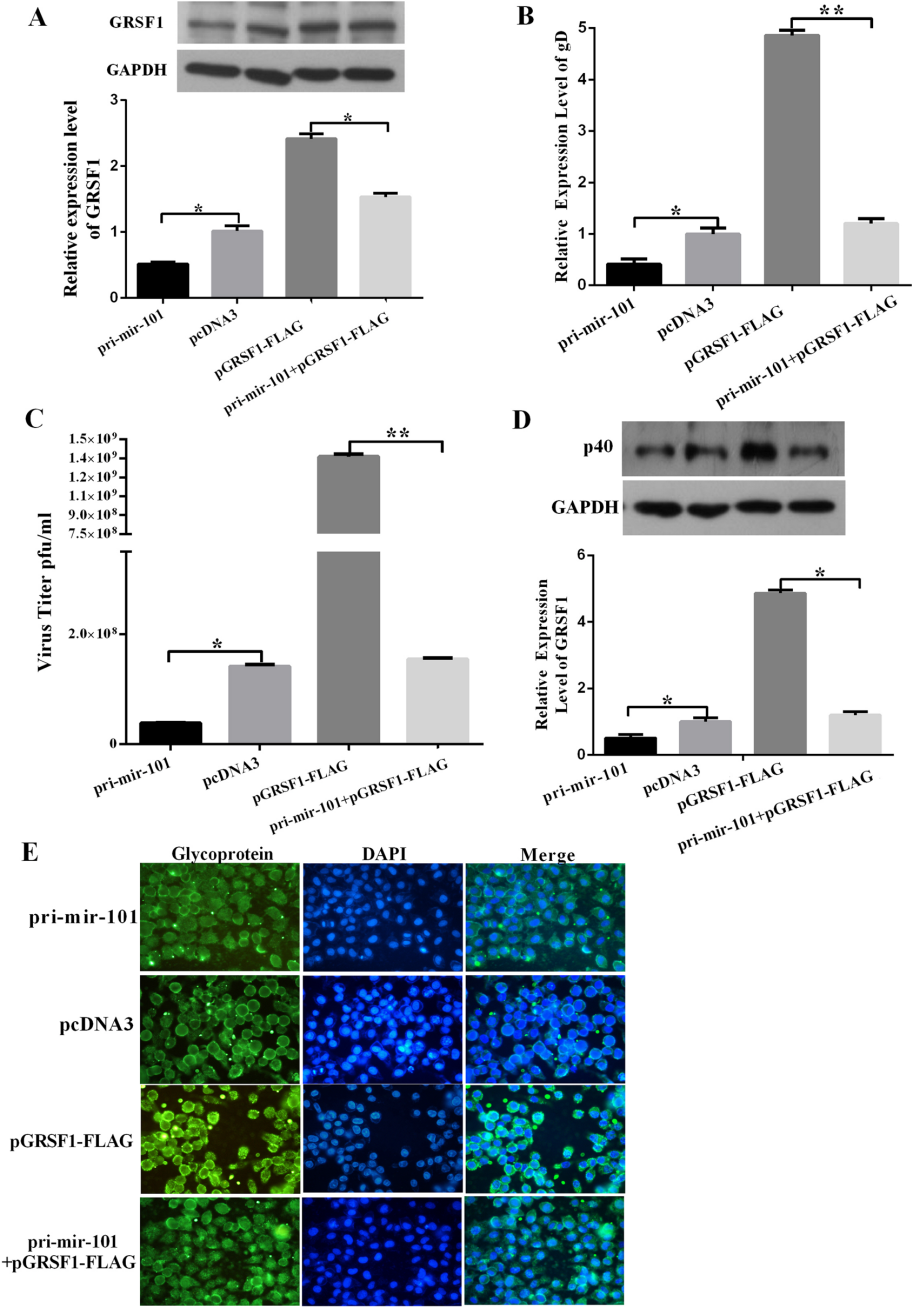
Overexpression of GRSF1 rescues the replication of HSV-1 that was repressed by pri-mir-101 in HeLa cells. (**A**) The overexpression of GRSF1 rescues the replication of HSV-1 that was repressed by pri-mir-101. HeLa cells were transfected with pcDNA3, pri-miR-101, or GRSF1-FLAG or were co-transfected with GRSF1-FLAG and pri-mir-101. GRSF1 protein levels were detected by Western blot. GAPDH protein was used as an endogenous control, and the relative quantity of GRSF1 protein is shown. (**B–D**) The overexpression of GRSF1 rescues the repression of HSV-1 replication that was caused by pri-mir-101. HeLa cells were transfected with pcDNA3, primiR-101, or GRSF1-FLAG or were co-transfected with GRSF1-FLAG and pri-mir-101. (**B**) The expression level of the gD gene was detected by qPCR. (**C**) Viral plaques in HeLa cells and (**D**) Western blot demonstrating HSV-1 replication in the presence of p40; GAPDH protein was used as an endogenous control. (**E**) Immunofluorescence was used to detect HSV-1 replication using an HSV-1 membrane protein in HeLa cells. *P < 0.05.

