# Supplementary material for: ICP4-induced miR-101 attenuates HSV-1 replication
Source: Sci Rep. 2016 Mar 17;6:23205. doi: 10.1038/srep23205 (PMC4794718; doi:10.1038/srep23205)
Supplement: Supplementary Information [file srep23205-s1.pdf]

ICP4-induced miR-101 attenuates HSV-1 replication

Xianling Wang[1¶], Caifeng Diao[1¶], Xi Yang[1], Zhen Yang[1], Min Liu[1], Xin Li[1], Hua Tang[1]*

1 Tianjin Life Science Research Center and Department of Pathogen Biology, School of Basic Medical Sciences, Tianjin Medical University, 22 Qi-Xiang-Tai Road, Tianjin 300070, China

***** Corresponding author

[¶]These authors contributed equally to this work

Correspondence and requests for materials should be addressed to H.T. (Tel & Fax: +86 22 23542503 E-mail: htang2002@yahoo.com; tangh@tmu.edu.cn)

Supplemental Data

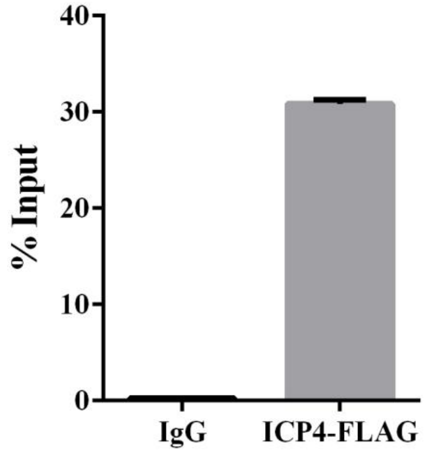

Figure S1. An immunoprecipitation of ICP4 using anti-FLAG and IgG from HeLa cells, which allows for the identification of RCL1/miR-101 promoter fragment. The DNA level of promoter fragment was detected by qPCR.

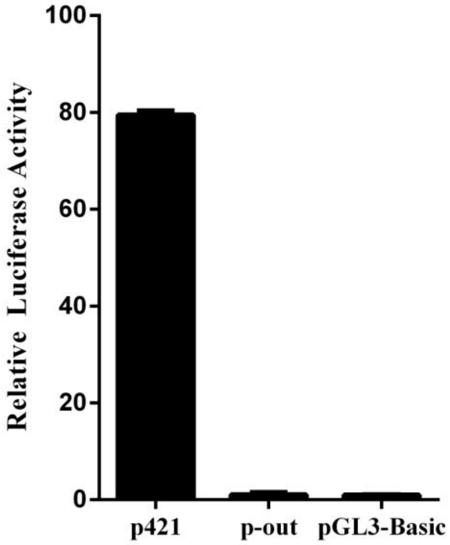

Figure S2. Luciferace activity of RCL1/miR-101 promoter deleted of ICP4 bind site. HeLa cells were transfected with promoter fragments (p421 has the highest promoter activity, p-out stand for the promoter deleted the binding site (59bp), pGL3-Basic as a control pkasmid) for 48h and determined by luciferase activity assays. Data are normalized against the vector

control and error bars present as means ±SD (n=3).

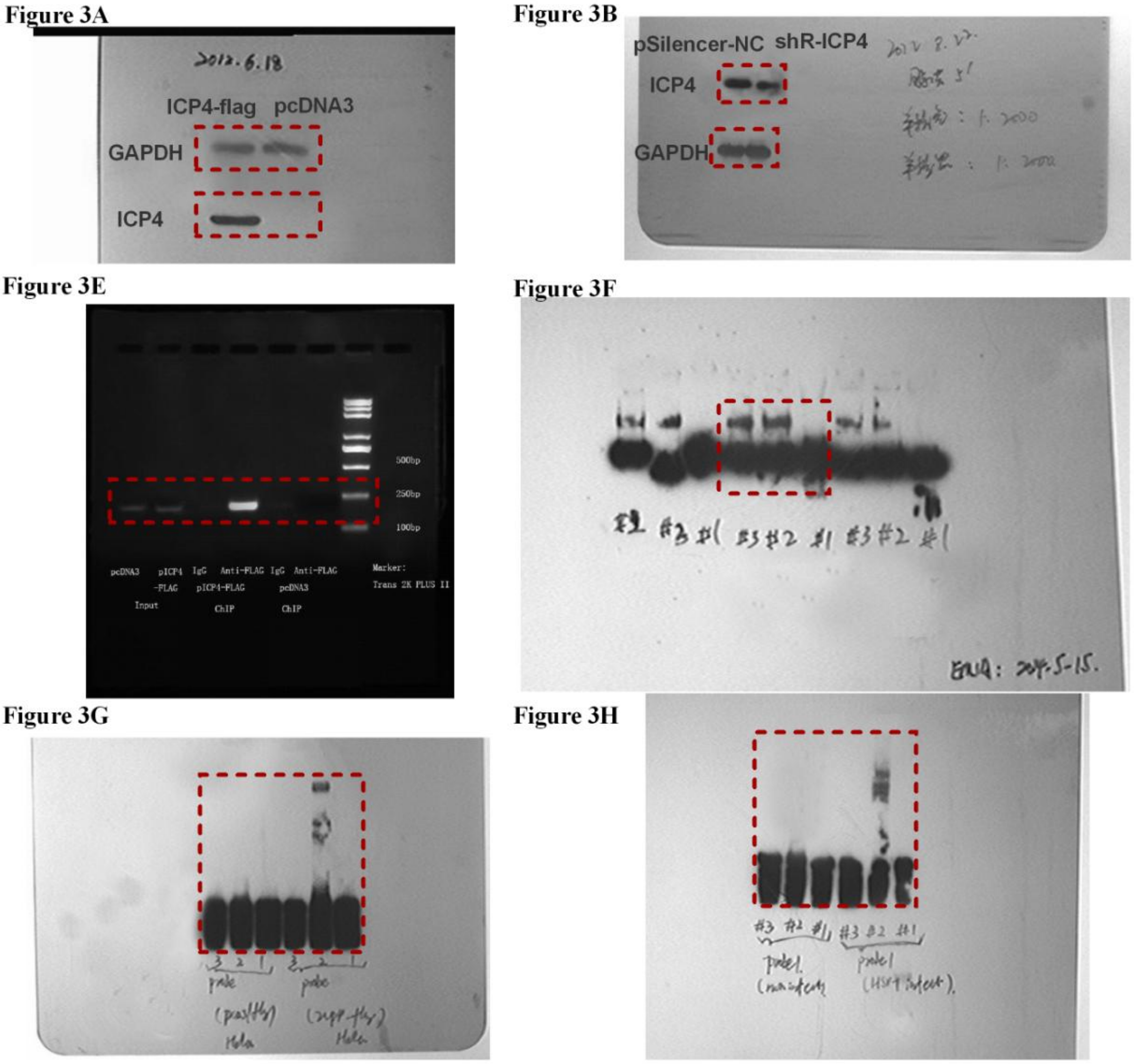

Figure S3. Uncropped, unprocessed images of blots and gels.

Figure 4D

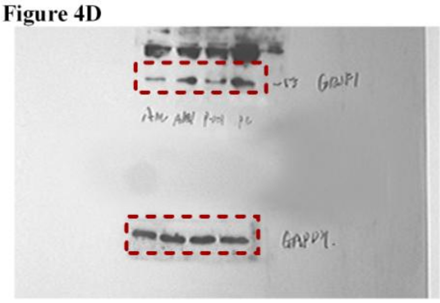

Figure 4F

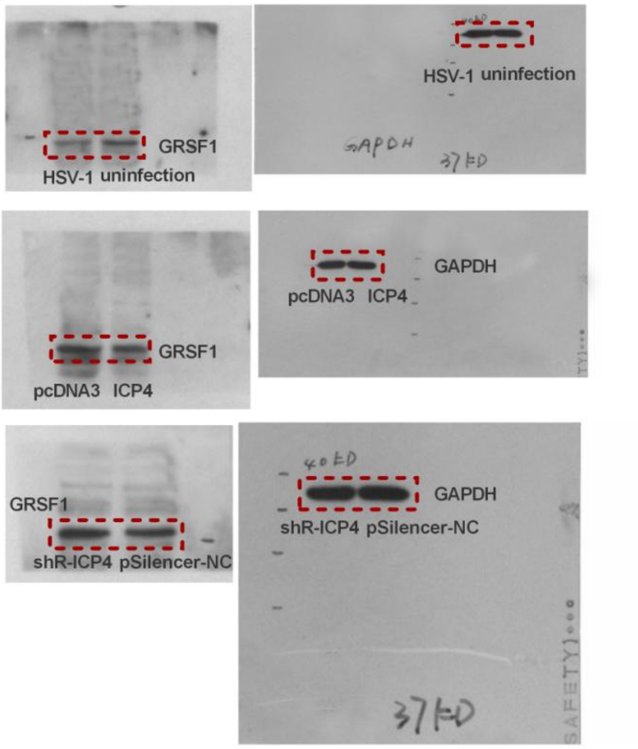

Figure 5A

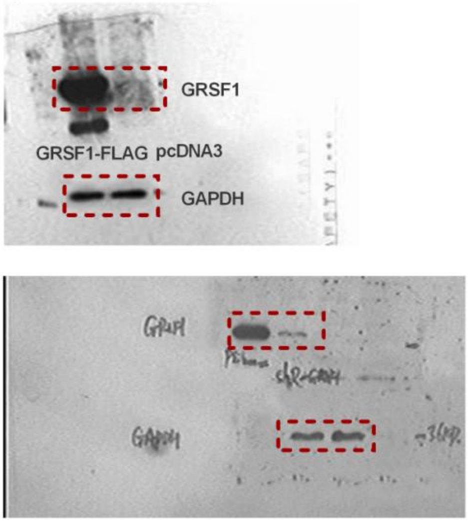

Figure S4. Uncropped, unprocessed images of blots and gels.

Figure 6A

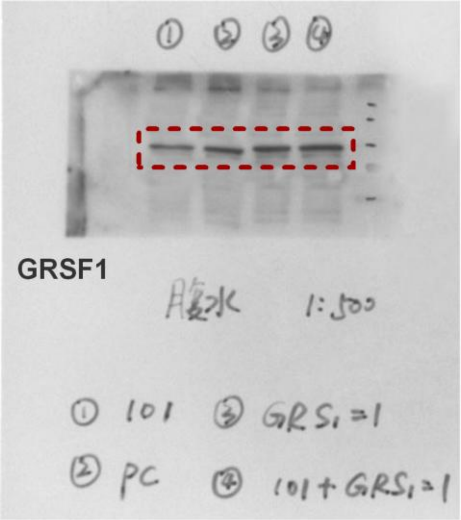

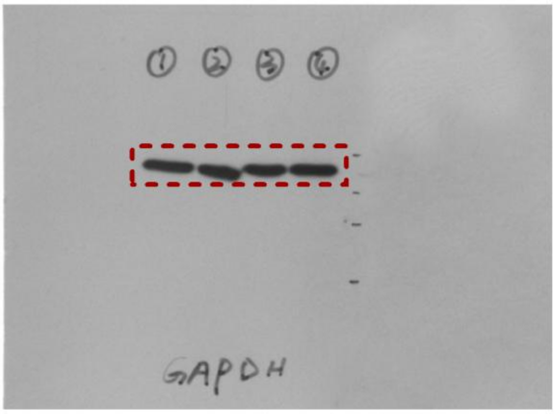

Figure 6D

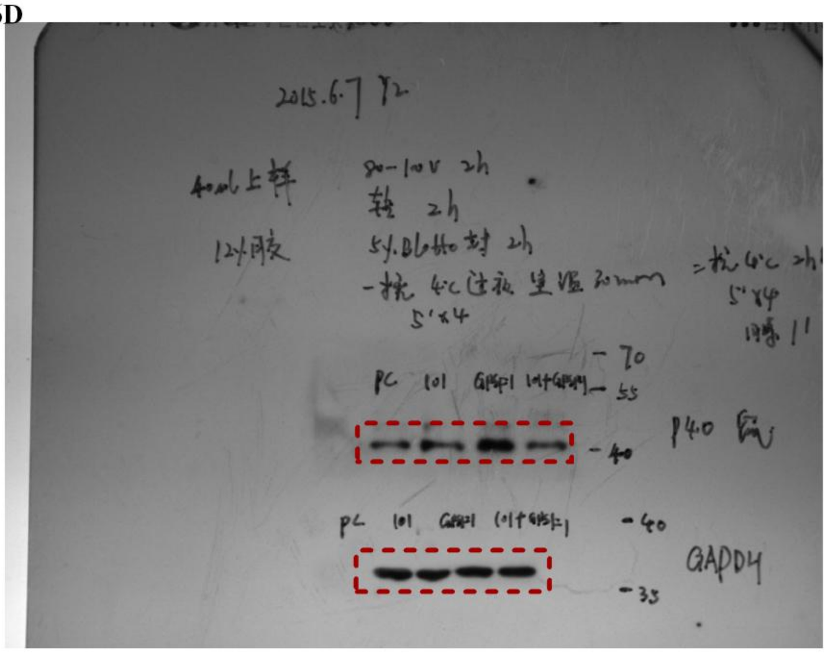

Figure S5. Uncropped, unprocessed images of blots and gels.

Figure5E

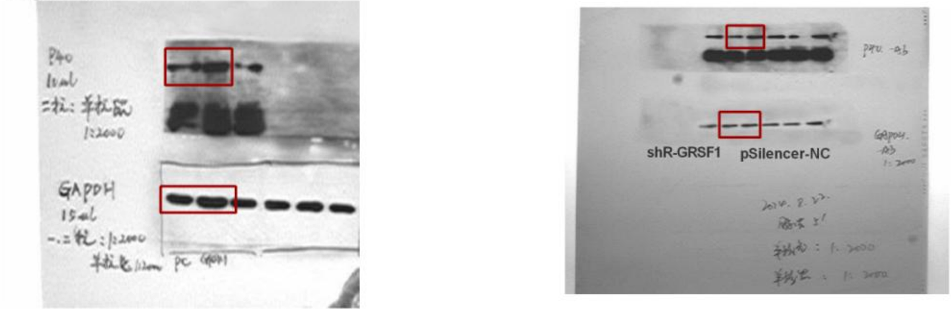

Figure 7A

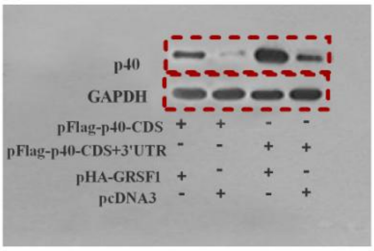

Figure 7B

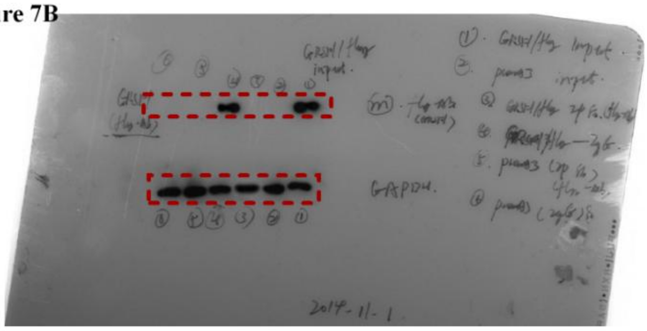

Figure 7C

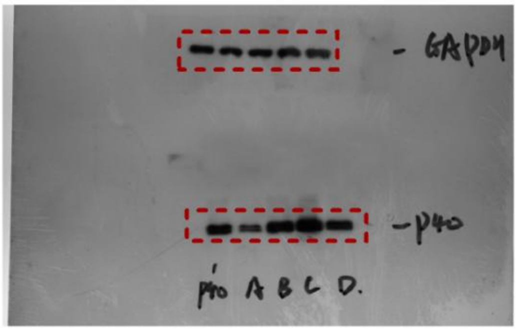

Figure 7E

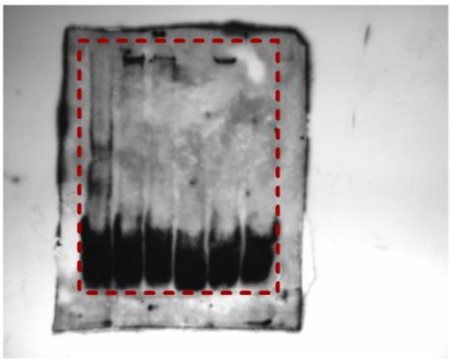

Figure S6. Uncropped, unprocessed images of blots and gels.

Figure 7D

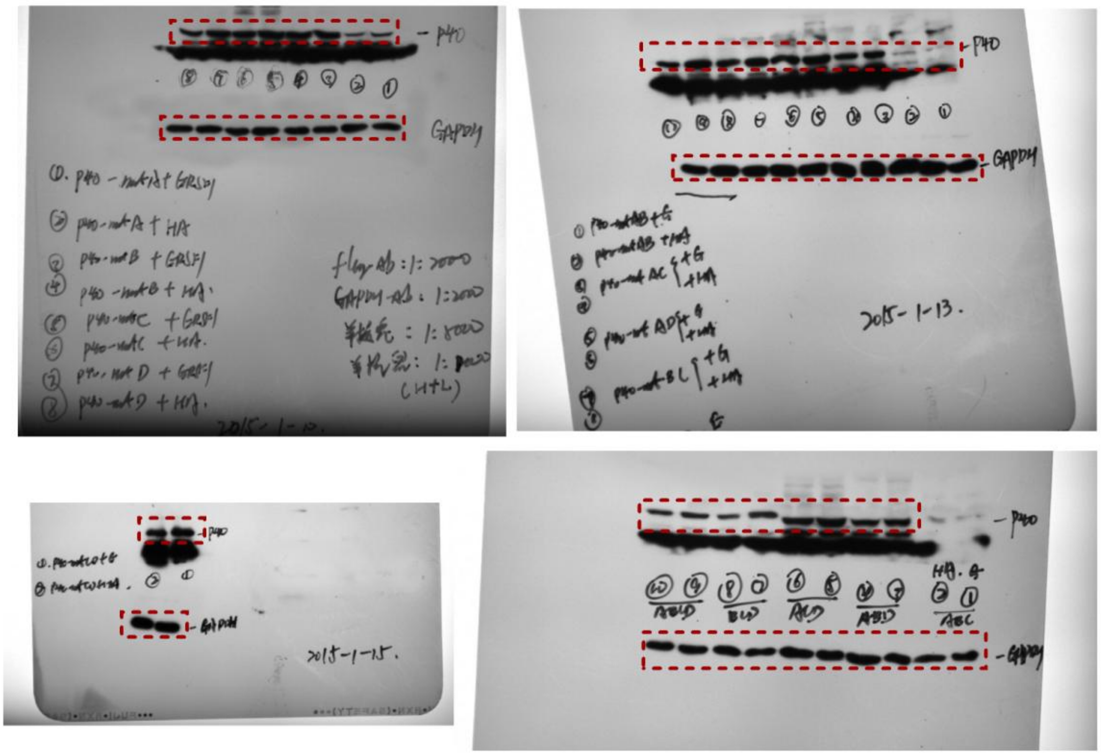

Figure S7. Uncropped, unprocessed images of blots and gels.

Table S1. Primer Sequence in this study.

Primer	Sequence
pGL3-p1060-S	5' GGGGTACCAACTCCCACCGCTTTCAG3'
pGL3-p1060-AS	5' GGAATTCGGAGAGACTAGACACCGGGTCAGAG3'
pGL3-p421-S	5' CGCGGTACCTAGCATCTTCACCCCAGTAG 3'
pGL3-p421-AS	5' GGAATTCGGAGAGACTAGACACCGGGTCAGAG3'
pGL3-p334-S	5' CGCGGTACCTAGCATCTTCACCCCAGTAG3'
pGL3-p334-AS	5' ATAGAATTCTTCGGGCCACGCCGCTATTG3'
ICP4-S-ER	5' CGGAATTCATGGCGTCGGAGAACAAGCAG3'
ICP4-AS-XI	5' ATAGCCTCGAGTTACAGCACCCCGTCCCCCTC3'
GRSF1-3’UTR-Top	5' AATTCAGATGCGAAATCACTGTACTGTAAGCTTC3’
GRSF1-3’UTR-Bot	5' TCGAGAAGCTTACAGTACAGTGATTTCGCATCTG3'
GRSF1-3’UTR-Top-mut	5' AATTCAGATGCGAAATGAGTCTAGACTAAGCTTC 3'
GRSF1-3’UTR-Bot-mut	5' TCGAGAAGCTTAGTCTAGACTCATTTCGCATCTG3'
CRSF1-RI	5' ACGGAATTCGAGTCCATGGCCGGCACGCGC3'
CRSF1-XhoI	5' GCAGCACTCGAGGCTTTTCCTTTTGGACATGAGTTCAGGAAC3'
shICP4-Top	5' GATCCAACAGCAGCTCCTTCATCACCCTCGAGGGTGATGAAGGAGCTGCTGTTTTTTTG3'
shICP4-Bot	5' AATTCAAAAAAACAGCAGCTCCTTCATCACCCTCGAGGGTGATGAAGGAGCTGCTGTTG3'
shGRSF1-Top	5' GATCCATGAGGATATTCAACCCATGACACTCGAGTGTCATGGGTTGAATATCCTCATTTTTTGA3'
shGRSF1-Bot	5'AGCTTCAAAAA ATGAGGATATTCAACCCATGACACTCGAGTGTCATGGGTTGAATATCCTCATG3'
miR-101 RT primer	5' TCGTATCCAGTGCAGGGTCCGAGGTGCACTGGATACGACTCATACAG3'
pre-miR-101-1-RT	5' TGCCATCCTTCAGTTATC3'

pre-miR-101-2-RT	5' ACCACCATTCTTCAGTTATC3'
U6 RT primer	5' GTCGTATCCAGTGCAGGGTCCGAGGTATTCGCACTGGATACGACAAAATATGGAAC3'
OligodT primer	TTTTTTTTTTTTTTTT
miR-101-Fwd	5' GCCCGCTCTTTGGTTATCTAG3'
U6-Fwd	5' TGCGGGTGCTCGCTTCGGCAGC3'
Reverse	5' CCAGTGCAGGGTCCGAGGT3'
pre-mir-101-1-S	5' TGCCCTGGCTCAGTTATCAC3'
pre-mir-101-1-AS	5' TGCCATCCTTCAGTTATCACAG3'
pre-mir-101-2-S	5' ACTGTCCTTTTTCGGTTATCATG3'
pre-mir-101-2-AS:	5' ACCACCATTCTTCAGTTATCACAG3'
RCL1-S	5' GTGAAGAACTCAAGGGTGGG3'
RCL1-AS	5' AAGCAAGTGGCTATTCTGT3'
β-actin-S	5' CGTGACATTAAGGAGAAGCTG3'
β-actin-AS	5' CTAGAAGCATTTGCGGTGGAC 3’
GRSF1-qPCR--S	5' GTCCTCTGCCCTGAACAGCCCTTA3'
GRSF1-qPCR-AS	5' CCAATTCAATGTGAGTGTCTTCCG3'
ChIP-S	5' TAGCATCTTCACCCCAGTAG3'
ChIP-AS	5' GCTGCCATTGGTCCGTAGTG3'
3’biotin-probe	5' AGCATCTTCACCCCAGTAGTTGCCGTAAGTAGTAATGCTAAGAATCCGGCACGTCGTAG3'
Unlabeled-probe	5' AGCATCTTCACCCCAGTAGTTGCCGTAAGTAGTAATGCTAAGAATCCGGCACGTCGTAG3'
gD-qPCR-S	5'CGCTTGGTTTCGGATGGGA3'
gD-qPCR-S	5'CTTCACGAGCCGCAGGTA3'
